# Assessment of exposure to pesticides and the knowledge, attitude and practice among farmers of western Bhutan

**DOI:** 10.1371/journal.pone.0286348

**Published:** 2023-05-30

**Authors:** Adeep Monger, Kiran Mahat, Dorjee, Namgay Om, Pooja Mongar, Tshering Dorji, Sonam Jamtsho, Karma Wangdi, Chador Wangdi, Thinley Jamtsho, Vishal Chettri

**Affiliations:** 1 Royal Centre for Disease Control, Thimphu, Bhutan; 2 National Plant Protection Centre, Ministry of Agriculture and Forest, Thimphu, Bhutan; 3 Department of Public Health, Ministry of Health, Thimphu, Bhutan; CPRI: Central Potato Research Institute, INDIA

## Abstract

An estimated 69% of the population of Bhutan is engaged in agriculture. Farmers are exposed to a wide variety of pesticides during the preparation, transport, storage, mixing and application of pesticides posing a significant health risk. A controlled cross-sectional study of farmers in selected sites of Bhutan was conducted to characterize the level of exposure to pesticides and assess their knowledge attitude and practice on the safe handling of pesticides. A total of 399 participants were enrolled in the study comprising of 295 exposed farmers and 104 healthy and unexposed controls. A structured investigator administered questionaries was used to assess their Knowledge, Attitude and practice, and their blood samples were taken for measuring Acetyl Cholinesterase enzyme activity level. There was a significant difference between the Acetyl Cholinesterase enzyme inhibition of exposed and non-exposed control groups observed in the study (*P* < 0.001). Of the total of 295 farmers, 62 (21.01%) had severe enzyme inhibition of >30% as compared to the unexposed group. Safety practices of handling pesticides were low. The most common symptoms self-reported were headache (OR 1.08, 0.60–1.93) and neurological problems like forgetfulness, lack of concentration (OR 1.12, 0.50–2.48) and increased tiredness (OR 1.075, 0.52–2.19) that were significantly associated with the enzyme inhibition. In addition, we record a very low level of knowledge (17.0%), a fair attitude (63.0%) and poor practice (35.0%) on the safe handling and management of pesticides. This pilot study provides indication of exposure to pesticides in the selected sites of the country. Furthermore, it provides evidence for public health interventions by identifying the exposure patterns and pathways of individuals most at risk in the farming communities of the country. Surveillance and bio-monitoring programs are deemed necessary.

## Introduction

Pesticides are substances that destroy, repel or attack pests and include: herbicides, insecticides, fungicides and rodenticides. Exposure to pesticides, short and long term/dose dependent effects is associated with various unintended adverse effects on humans and the environment [1]. More specifically, exposure is associated with development of wide spectrum of diseases such as cancer, endocrine disturbances, asthma, hypertension, diabetes, allergies and also developmental toxicities like miscarriage and infertility [2–4].

In rural Asia and China, the World Health Organization’s (WHO) class I and II organophosphates (OP) and carbamates are the most frequently involved pesticides causing accidental, unintentional and occupational poisoning [5, 6]. These OP and carbamate pesticides affect the nervous system by inhibition of the acetylcholinesterase enzyme (AChE) causing poisoning to agriculture workers and children [7].

In Bhutan, an estimated 69% of the population is engaged in agriculture [8]. A range of insecticides, fungicides and herbicides are imported and widely used in the country. And Bhutanese farmers are likely to be exposed to a wide variety of pesticides during the preparation, transport, storage, mixing and application of pesticides [9]. Moreover, there are unsubstantiated reports indicating the use of pesticides without the recommended safety measures by farmers.

Currently, very little is known about the extent of pesticide exposure among farmers and the awareness levels about the potential toxicity of inappropriate handling of pesticide and use of personal protective equipment (PPEs) while handling pesticides. Although the government emphasizes organic agriculture, the use of pesticides has been increasing due to frequent outbreaks of pests and diseases such as fall armyworm, rice blasts and chili blight [10]. Notably, there is a steep rise in diseases such as cancer, hypertension and diabetes that are of increasing public health concern among the Bhutanese population [11] which maybe attributed to chemicals.

Many developing countries implement bio-monitoring and surveillance programs intended to protect farmers from the adverse effect of pesticide exposure, [12, 13]. Monitoring programs are undertaken to detect early physiological changes before resulting in reversible or irreversible diseases and illness. The globally accepted monitoring programs include the measurement of peripheral cholinesterase enzymes; the erythrocyte and serum cholinesterase [14]. These are the approved surrogate biomarkers for exposure of OP and carbamate pesticides [7]. The inhibition of the AChE enzyme is the early event of poisoning by OPs and carbamates before the onset of severe neurological conditions [15]. Such surveillance and bio-monitoring programs are unavailable in the country.

Therefore, considering the likely exposure to pesticides and its detrimental health risk of pesticides to farmers in Bhutan, it is important that exposure assessment is conducted. So that biological monitoring programs are initiated for early detection and intervention to ensure appropriate interventions for a healthy and productive population. This will also assist in developing evidence-based health policies, formulate treatment guidelines for pesticide related diseases, establishing valuable baseline data and to implement a long-term prognosis. Therefore, this study aims to determine level of pesticide exposure, identify the associated risk factors and assess the knowledge, attitude and practice on proper handling and safe management of pesticides.

## Methodology

### Study type and settings

**Fig 1:**
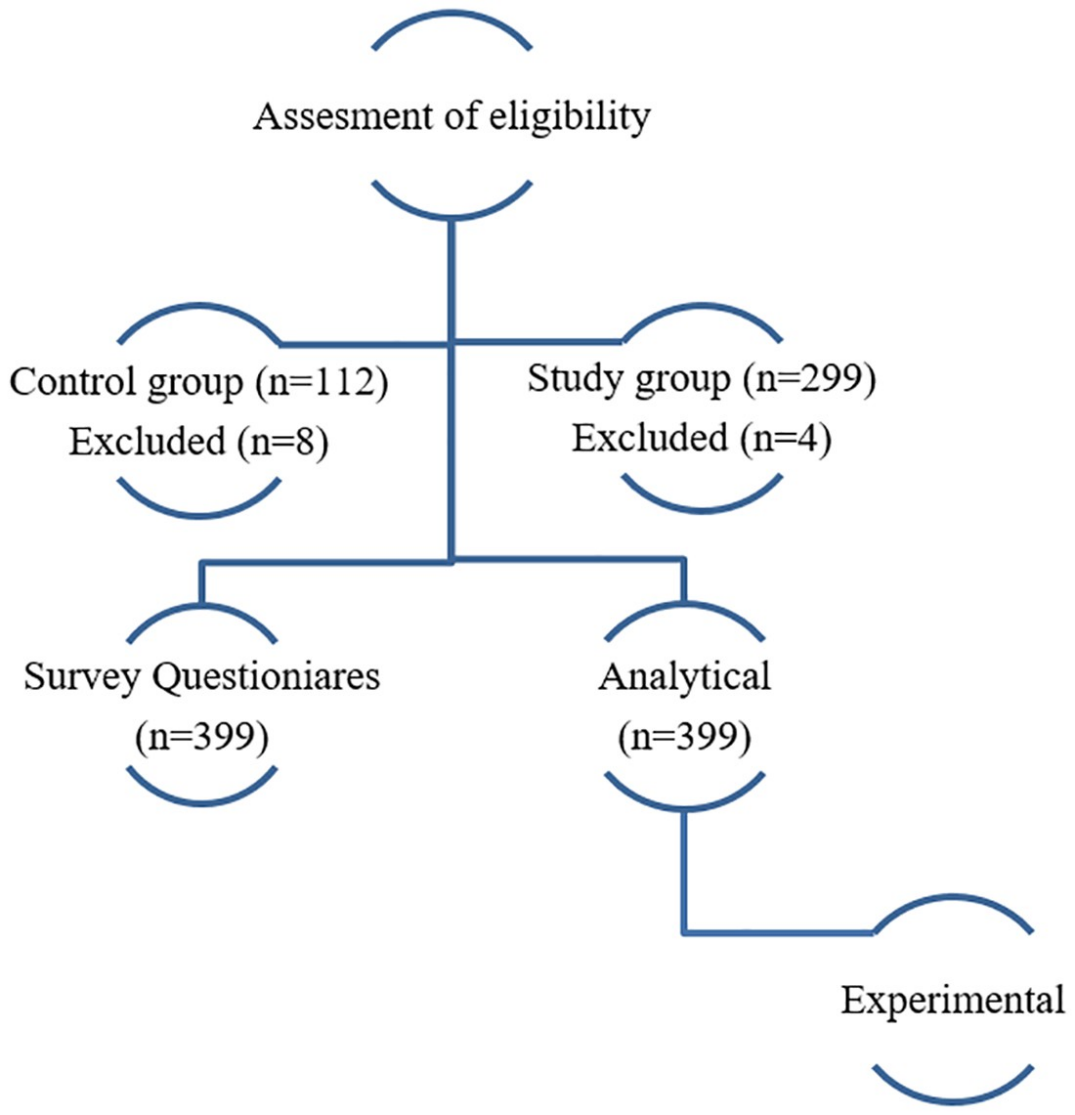
Sampling design used in the study. N = number of volunteers.

**Fig 2:**
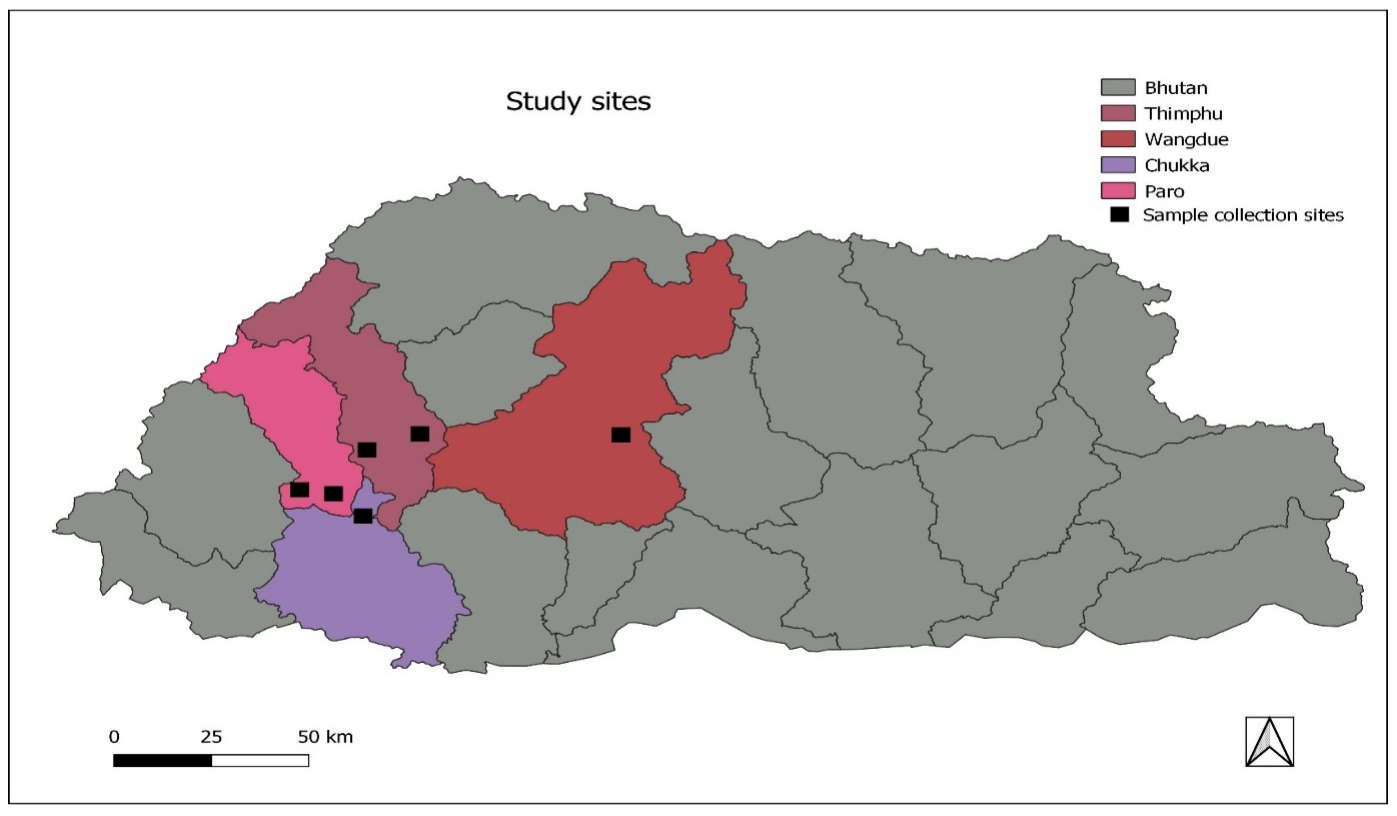
Sample sites for the study.

The study employed the prospective observational approach to assess exposure via the comparative inhibition of AChE enzymes in farmers that handled pesticides and the control group for comparison (i.e. individuals not directly exposed to pesticides). The Fig 1 shows the sampling design used in the study. From the dataset maintained by National Plant Protection Centre, a total of nine villages from four districts (Fig 2) that use the maximum pesticides in the country were sampled for the study [16]:

1. Tshongkha/Dawakha(Dogar, Paro)
2. Wanakha (Naja, Paro)
3. Susuna (Naja, Paro)
4. Shemagangkha (Chapcha, Chukha)
5. Dorgon, Dantak area (Chapcha, Chukha)
6. Bjemina (Mewang, Thimphu)
7. Hongtsho (Chang, Thimphu)
8. Yusipang (Chang, Thimphu)
9. Phobjikha (Phobji, Wangdue)

### Sample size

The sample size was determined at confidence interval level of 95% with a desired margin of error of ± 5% with population proportion of 50% using epi info statcalc version 7.2.4.0 [17]. The target population of famers (i.e. pesticide handlers and control) from sample villages of four dzongkhags were randomly selected from the list of households provided by the government officials. A total of 295 farmers handling pesticides were involved in the study.

Inclusion criteria for farmers from the sample household

1. Those farmers who have handled pesticides
2. All farmers consenting to participate

Exclusion criteria of farmers from the sample household

1. Those farmers who declined and did not consent to participate
2. Who were not involved directly in handling pesticides or not working in pesticide related activities

Control group were purposely selected based upon the following criteria:

1. None of the participants had been recently exposed to pesticides occupationally
2. Age range (>18 and above) and other demographic variables (Example- civil servants, private employees and shopkeepers in the nearby premises of the farming households/villages were included)

Of the total of 112-control group, only 104 samples were used for analyses as they were rejected due to the pathologies and other exclusion criteria used for the sampling inclusion and exclusion criteria above.

### Study tool

An investigator-administered questionnaire was used to collect demographic information, risks, KAP on risks, proper handling, and safe management of pesticides. Sample farmers were asked about safe handling of practices, and symptoms associated with pesticide exposure. For the control group, only the demographic variable, occupation, comorbidities and whether they handled pesticides or not were asked to select samples for the study. Finally, blood samples were collected from all the consenting and qualifying participants to test for AChE inhibition assay.

### Blood sample collection and testing

From each sample participant, 5 ml of blood samples was collected in vacutainer. Prior to the blood collection, the puncture site was thoroughly cleaned twice with 70% alcohol and proper infection control practices were followed. A clean setup for the location of the blood collection room was maintained and allocated in the field area. Blood samples were stored temporarily on-site. The required temperature of 4^0^ Celsius was maintained by keeping the samples in the cold chain box, transported to the lab and maintained at -20°C until analyzed.

The test for AChE inhibition was performed with Ellman method [18] as per the manufacturer’s instruction in the leaflet. Briefly, whole blood samples were diluted 40-fold in assay buffer (5μl of blood in 195 μl of assay buffer). Acetylthiocholine reaction mix and Acetylcholinesterase standard solution were prepared accordingly. The serial dilutions of AChE standard from 0 to 1000 mU/ml were prepared to construct a calibration graph for the quantification of the AChE enzymes in the samples. A 50 μl of Acetylthiocholine Reaction Mix was added to each well of the Acetylcholinesterase Standard, Blank Control, and Test Samples. The reaction tubes were incubated for 10–30 minutes at room temperature, protected from light. Finally, the absorbance increase was monitored using a colorimetric microplate reader at 405–415 nm (max abs at 412 nm).

### Determination of cut-offs for AChE inhibition

The reference value for blood cholinesterase levels has been not established in the country yet. Also, the baseline value of the study population was not measured in the study. Therefore, the AChE levels were compared with the normal unexposed population range. The benchmarks for cholinesterase in whole blood were determined from the enzymatic activity of the enzymes in the control group stratified by gender. To classify, the inhibition in the case/exposed groups to OP pesticides, an inhibition value of 30% was used accordingly to the US EPA [19].

### Questionnaire

The study questionnaire was adopted and modified from studies elsewhere [20–22]. Briefly, the questionnaire consisted of 53 KAP questions in total and was divided into seven section:

1. Section A described the respondent’s background information. Questions were based on gender, age, and their literacy level
2. Section B questions included number of farming years, types of crops grown, and types of pesticides used
3. Section C assessed the identification of poisoning risk factors which included questions on their pesticides spraying techniques, storage, management, and their daily practices of pesticides
4. Section D included question on knowledge on safe handling of pesticides. Safe use of PPE, use of right ratio of pesticides, pesticides application time and if they have received any training on safe handling of pesticides before or not were asked
5. Section E assessed on attitude of farmers on safe handling of pesticides. Their attitudes towards use of PPE (gloves, mask and apron), their self-hygiene and care after handling pesticides were asked
6. Section F was based on practice of safe handling of pesticides. Questions were on their proper use of PPE or not, if they ate food/drank at the site of practice, if they take bath after the application of pesticides and if they read the labels before application of any pesticides
7. Section G were questions on prevalence of exposure signs and reported symptoms among the study group. About thirteen signs and symptoms related to pesticides were asked with an option “yes” or “No”.

To assess the reliability, questionnaire was pre-tested beforehand among the experts from National Plant protection centre and public health officials. The Cronbach’s Alpha value for knowledge, attitude and practice sections showed an acceptable result of 0.62. Table 1 shows the cut-off values and classification of KAP.

**Table 1:** Classification of knowledge, attitude, and practice.

Score Values	Knowledge	Attitude	Practice
Total >80%	High	Concern	Good
61–80%	Moderate	Neutral	Fair
≤60%	Low	Not concern	Poor

### Data analysis

The data was entered in forms created with Epi info version 7 and extracted and analyzed using both Epi info version 7 and IBM SPSS Version 22. The prevalence of AChE inhibition was expressed in the form of percentages. Mean or median (wherever applicable) were used to quantify levels of AChE inhibition results stratified by key variables related to exposure characteristics. Univariable analyses were used to examine the unadjusted association of various independent variables with elevated inhibition levels.

An independent samples *t-*test was conducted to compare difference in AChE inhibition between the exposed and the control group. Similarly, difference in AChE levels between different variables were compared using an independent samples *t*-test analysis. A one-way ANOVA was conducted to test the difference in AChE levels between locations, age groups, farming experience in years. When a significant difference was detected by the ANOVA, treatment means were compared using Tukey’s HSD test. Differences in treatment means were considered statistically significant at the *p* = 0.05 level. The data were analyzed using IBM SPSS Version 22 and results are presented as means ± SD.

### Ethics approval

Ethical approval was sought from the Research Ethics Board of Health in Bhutan Ref. No. REBH/Approval/2022/015. Written informed consent was obtained by getting both the signature on the approved consent form from the eligible farmers prior to interview and that of the person obtaining consent. Only those consenting were interviewed by the investigators. Administrative clearance from each village/districts was obtained prior to starting the study.

## Results

### Socio-demographic characteristics

A total of 399 participants were enrolled for the study, of which 295 (73.9%) were exposed and 104 (26.0%) were unexposed control group. There were 214 (53.63%) males and 185 (46.36%) of the females. Among the exposed farmers group, 55.2% (n = 163) were males and 44.7% (n = 132) were female.

The mean age of male was 45.78 ± 13.28 and female was 42.89 ± 12.43. Between the different age categories of respondents, the majority of the respondents (n = 120) were in the age group of 35–49 years and the least was observed in the age category of 18–24 years (n = 11). It was found that 48.5% (n = 145) of the respondents were literate while 51.5% (n = 154) were illiterate. The socio-demographic characteristics table is listed in Table 2.

**Table 2:** Socio-demographic characteristics of the exposed participants.

Variables	n	%
Gender		
Male	163	55.2
Female	132	44.7
Age (Years)		
18–24	11	3.72
25–34	64	22.07
35–49	120	40.6
50–59	61	20.6
> 60	39	13.2
Literacy		
Literate	144	48.8
Illiterate	151	51.1

During the study period, the majority of farmers had been farming between 21–30 years (22.71%) while the least was between 1–5 years (10.51%). Among the types of crops grown, the majority (87.2%) grew potatoes, whereas the least grown was rice (0.3%). It was found that almost all the farmers included in the study use pesticides in their farms. The most common pesticides used were cypermethrin (58.19% (n = 174)) followed by mancozeb (22.07% (n = 66)) of famers used cypermethrin, whereas only 0.33% (n = 1) used malathion. The Table 3 represents the farm information.

**Table 3:** Farming characteristics.

Variables	n	%
No. of farming years		
1–5 years	31	10.51
6–10 years	35	11.86
11–15 years	52	17.63
16–20 years	49	16.61
21–30 years	67	22.71
> 30 years	61	20.6
Types of crops grown		
Rice	1	0.3
Maize	28	9.4
Potatoes	261	87.2
Chilies	29	9.7
Green vegetables	165	55.2
Others*	150	50.2
Types of pesticides used		
Cypermethrin	174	58.19
Mancozeb	66	22.07
Metribuzin	86	28.76
Glyphosate	62	20.74
Chlorpyrifos	3	1
Malathion	1	0.33
Others**	160	53.51

*others includes: cabbage, beans, radish, saag

**others includes sunrice,butachlor, sulphala, urea

### Prevalence of blood AChE inhibition

The overall mean of AChE levels of the study participants was 673.9 ± 144.4 mU/ml. A significant difference between the AChE enzymatic activity of exposed and non-exposed control groups (652.01 vs 733.68) was observed in the study (*p* < 0.001). Male exposed farmers had a slightly lower AChE level (642.7 ± 153.0 mU/ml) than exposed female farmers (663.7 ± 141.6 mU/ml), however this difference was not significant (*p <* 0.216).

Furthermore, the benchmarks for the total AChE inhibition in whole blood were obtained from the mean values of the control group (n = 105) for comparison. The overall activity of the AChE enzyme in the control group corresponded to 733.68 mU/ml, and 703.87 mU/ml in males and 755.96 mU/ml in females. Therefore, a 30% inhibition of AChE cut-offs was adopted as 513.0 mU/mL in all participants, 492.71 mU/ml in males and 529.18 mU/ml in females respectively.

Of the total of 295 farmers, 62 (21.01%) had severe AChE inhibition of > 30% as compared to the unexposed group. The prevalence among the exposed farmer population was 20.60% in males and 20.76% in females. Table 4 represents the data. Male exposed farmers have a slightly lower AChE level (642.7 ± 153.0 mU/ml) than exposed female farmers (663.7 ± 141.6 mU/ml), however this difference was not significant (*p <* 0.216). Similarly, no difference in AChE levels were observed for farmers who were literate compared to those who were not (*p >* 0.811).

**Table 4:** Prevalence of AChE inhibition.

Groups	AChE levels (mU/mL)				Prevalence of inhibition			*p-value*
	Exposed		Unexposed (Control)					
	n	Mean ± SD	n	mean ± SD	cut-offs	n	%	
Overall	295	652.01 ± 148.25	104	733.68 ± 112.85	513.0	62	21.01	< 0.001*
Male	165	642.7 ± 153.02	49	703.87 ± 211.16	492.71	34	20.60	0.0021
Female	130	633.77 ± 141.67	55	755.96 ± 105.12	529.18	27	20.76	<0.00001*

*Significant at *p* <0.001; Statistical test ANOVA Correlation

**Fig 3:**
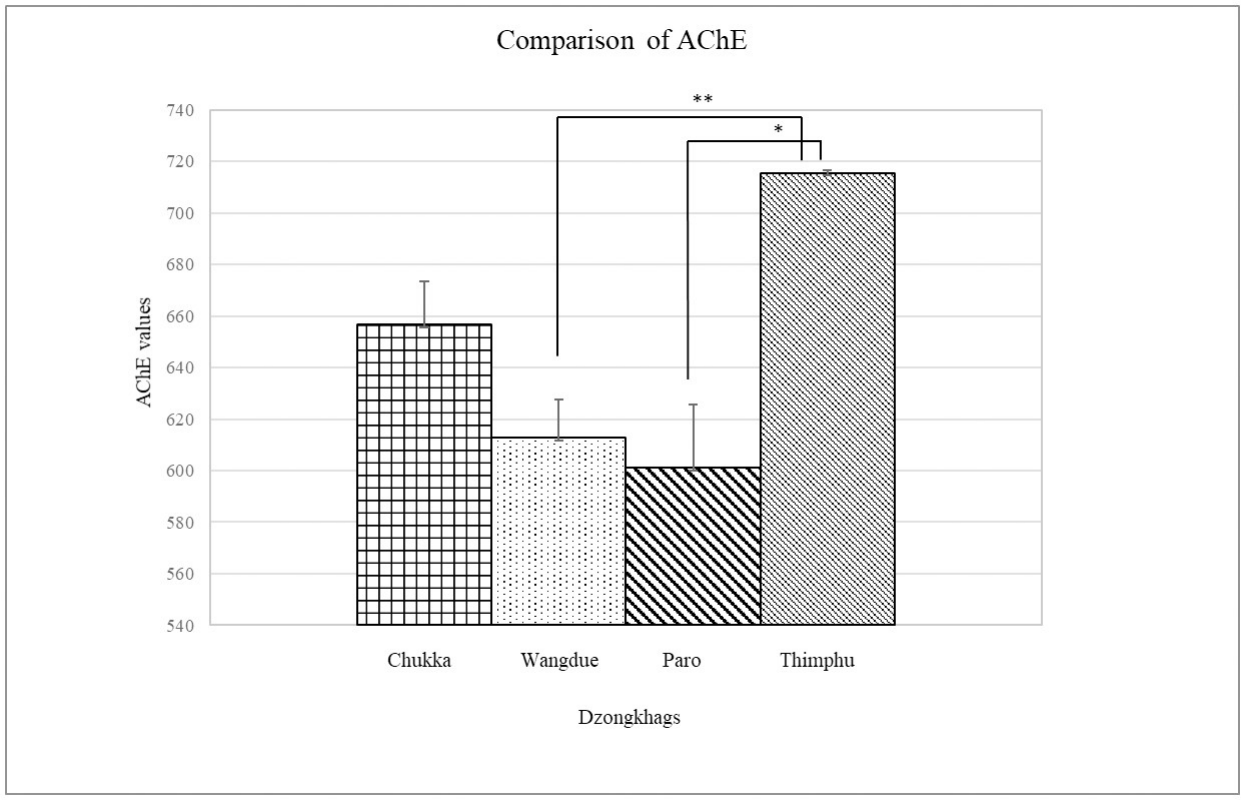
AChE level (mean ±SD) comparison between dzongkhags, with 95% confidence intervals.

A one-way ANOVA indicated a significant difference in AChE levels, for farmers in the exposed group, between different dzongkhags (*p <* 0.001) (Fig 3). Post-hoc comparison showed that AChE level for the exposed group in Wangdue and Paro were significantly inhibited compared to Thimphu, whereas Chhukha and Thimphu showed no such differences in AChE inhibition. Farmer’s age did not significantly influence AChE levels in the exposed group (*p >* 0.846). Similarly, farming experience in years also did not influence AChE levels in the exposed groups (*p >* 0.721), although farming groups in the 1–10 and 10–20 years showed a slight inhibition in AChE levels compared to those in 20 to 30 and 30 years and above farming experience (Table 5).

**Table 5:** Comparison of AChE level with working years.

Work duration (yrs)	N	Mean	Std. Deviation	Std. Error	95% CI for Mean		Min	Max
					Lower	Upper		
1–10	66	646.21	165.52	20.37	605.52	686.90	271.61	889.97
10–20	100	643.74	154.31	15.43	613.12	674.36	295.02	889.97
20–30	68	669.35	132.75	16.10	637.22	701.48	332.21	889.97
>30	61	652.49	136.21	17.44	617.61	687.38	291.76	889.97
Total	295	652.01	148.25	8.63	635.02	668.99	271.61	889.97

### Pesticide usage and poisoning risk factors

The main risk factors of poisoning identified in the study were farmers spraying and finishing whatever pesticides mixtures that they have made (82.6%) and disposing the empty containers along with the household garbage (40.0%). In addition, 34.0% of the farmers were routinely spraying pesticides regardless of whether they observe pests or not. Data is available in S1 Table.

### Prevalence of clinical symptoms associated with pesticide usage

The most common symptoms reported in our study by the farmers was headache (61.0%), forgetfulness/lack of concentration (61%) followed by burning/watery eyes (43.0%) and increased tiredness (36.9%) respectively (Table 6). A comparative analysis using chi square test was conducted in an attempt to determine the relationship between blood AChE level and subsequent clinical symptoms. Headache and forgetfulness/lack of concentration were significantly associated with the inhibition of the AChE enzyme (*p-value* 0.0146 and <0.0001).

**Table 6:** Prevalence of exposure signs and reported symptoms (n = 295).

Clinical symptoms	*n*	(%)	OR	95% CI		*p-value*
				Lower	Upper	
Headache	180	61.0	1.08	0.60	1.93	0.014**
Anxiety/nervousness/irritable/severe shyness	73	24.7	1.07	0.59	1.91	2.54
Forgetfulness/lack of concentration	180	61.0	1.124	0.50	2.48	<0.0001*
Burning/Watery eyes	127	43.0	0.50	0.23	1.07	6.51
Increase in salivation/Drooling	5	0.01	0.85	0.08	8.4	0.21
Nausea/vomiting/Diarrhea	42	14.2	0.833	0.355	1.95	0.11
Difficulty in breathing	32	10.8	0.35	0.152	0.80	4.70
Cough	64	21.6	7.48	3.21	17.41	0.10
Stomach cramps	49	16.6	0.641	0.284	1.44	2.64
Clumsiness/tremors	49	16.6	0.753	0.319	1.77	2.5
Muscle Aches	96	32.5	1.084	0.521	2.25	1.2
Increased tiredness	109	36.9	1.075	0.52	2.19	0.5
Skin rashes	36	12.2	1.059	0.36	3.04	0.14

*Significant at *p* **<0.001**,

**Significant at *p* <0.05

### Knowledge, attitude and practice of farmers

The questionnaire used for the assessment in the current study was pre-tested and adapted from previous studies conducted elsewhere [23, 24]. There were 7 knowledge assessment questions, four attitude questions and eight practice assessment questions (S2–S4 Tables). Previously published methods were used for calculating knowledge, attitudes and practices scores [24].

The present study shows that only 17% (n = 52) of the interviewed farmers had a high level of knowledge and majority (59%, n = 177) had low knowledge on the usage of pesticides (Table 7).

**Table 7:** Score distribution of KAP (n = 299).

Variables	Level	N	%
knowledge	High	52	17
	Medium	70	23
	Low	177	59
Attitude	Concerned	188	63
	Medium	0	0
	Not concerned	112	37
Practice	Good	74	25
	Fair	119	40
	Poor	104	35

The attitude assessment consists of four questions that could be answered by ‘strongly agree to strongly agree’ or being neutral. The study shows that, 63% (n = 188) of the respondents had concerned attitude and 37% (n = 112) of them are least concerned with right attitude while handling pesticides safely while working in the field. To know the practice methods by the farmers eight questions was administered responded either by never do this to always do this. The study shows that 25% were following good practice, 45 were following fair method and 35% were following poor practice method. We found a positive and significant correlation between Knowledge and Practice scores (r = 0.4, *p* <0.001) in this study. However, there was no significant correlation between practice and attitude (*p* <0.001).

## Discussion

This is the first ever study to assess the exposure to pesticides and the knowledge, attitude and practice regarding the safe handling and management of pesticides among the farmers in four Dzongkhags (regions) of Bhutan. The study exhibited a significant depression of AChE enzyme level among farmers as compared to the control. Neurological problems like headache, forgetfulness, lack of concentration and increased tiredness were associated with AChE inhibition. Moreover, the observation on safety practices of handling pesticides were low.

A statistically significant level of inhibition of AChE activity in farmers were reported low as compared to the control unexposed group (*p* < 0.001) giving a prevalence of 21.01%. This indicates that farmer’s depression of AChE enzyme is attributed to the pesticide exposure raising a concern to their health. Factors such as knowledge on the safe handling of pesticides and the farmer’s practice were directly linked. Similar studies elsewhere reported an inhibition of 4.6% in Brazil [25], 27.0% in Arusha, Tanzania [26], 68.1% in Thailand [27] and 50.6% in Ghana [28] who recognized pesticide exposure as the main contributing factor. However, there was disparity in the prevalence among the different regions which might be due to the exposure frequency of pesticides, types of pesticides used and the cut-off benchmarks values used in the current study.

In the present study, the AChE depression at 30% was selected to quantitatively estimate the exposure to pesticides which is the WHO’s biological index for individuals occupationally exposed to pesticides [19]. Nevertheless, in a clinical practice the initial baseline value of AChE should be collected before and compared after the handling of pesticides to determine the exposure as per the standard protocols elsewhere [12]. Exposure to pesticides in farmers occurs due to unsafe working practice such as not using proper personal protective equipment, having a limited knowledge on the safe handling and management of pesticides [29]. Therefore, advocacy on the proper use of pesticides, the risk associated to it and bio-monitoring programs are necessary.

A comparative analysis of AChE blood level with self-reported clinical symptoms between exposed and control indicated a statistically significant association in number of exposure symptoms reported. The farmers mostly complained of headache, burning/watery eyes and neurological problems like forgetfulness, lack of concentration and increased tiredness. Similar pesticide poisoning syndromes were reported by Khan et al [30] Prado et al [31] and Mwabulambo et al [26]. This is because OP and carbamate class of pesticides causes four main neurotoxic effects in humans; the cholinergic syndrome, the intermediate syndrome, organophosphate-induced delayed polyneuropathy and chronic organophosphate-induced neuropsychiatric disorders [32]. Hence, agreeing with the reported clinical symptoms by the exposed farmers in the present study. This signify that general health effects of pesticide exposure are real among the farmer communities in the western region of Bhutan.

The knowledge of farmers on proper use of pesticides was found to be low, although 48.5% of the respondents were literate. Interestingly, the attitude of study participants showed that almost two third of the respondents had concerned attitude. However, the practice of pesticide use in farming was at a moderate level. Only 50–59% of farmers used mask and gloves but there was no proper use of PPE. This finding is also in line with the studies about health risk assessment of farmers where 25% of farmers in Argentina, 41.8% in Ethiopia, high number of 72.5% in Morocco, 13.8% in Kermanshah, Iran and about 40–70% in South India don’t use PPE while applying pesticides [33–37]. Reasons for failing to use proper PPE were either it was too uncomfortable to wear or due to the hot weather [27], recommended use of expensive PPEs, locally unavailable, confusing illustration of PPE usage instructions [38]. In addition, in most developing countries use of inadequate protections is due to less income and household wealth, lack of training and information on the use [39]. However, despite the factors like cost, availability, thermic and mechanical discomfort for improper use of PPEs, it is not always an advisable effective prevention [40].

Regarding the application of pesticides, 41% of farmers spray pesticides in the morning or afternoon and avoid it during windy and rainy weather. In the present study, 73.3% of farmers changed clothes and 87.54% washed hands after their application of pesticides and 67.80% avoided consuming food and drinks while applying pesticides showing fair knowledge on good practice of pesticides. However, about 52.38% fail to read pesticides labels before its application. Likewise, similar studies in Argentina have also reported about 25% of farmers who don’t read labels before the application with following reasons; too complicated to understand or they acquire loose agrochemicals without labels, and some have reasoned as labels comes in non-native languages [33]. Therefore, it is important that the material safety data sheet/leaflets should be translated to native languages.

The knowledge and attitude towards pesticides are known to be linked to the proper practice of handling pesticides. In the current study, 51.5% of farmers were illiterate and more than 78% of farmers in country have not attended trainings or any education programs related to pesticides in their years of farming. As stated, less educated/uneducated populations might be at higher risk when using pesticides, possibly due to difficulties in understanding the labels and its safety procedures [41]. Aligned with our study, farmers in Nakhon Nayok, Thailand initially have reported low knowledge of appropriate handling of pesticides (22.3%) and practice of pesticides use (22.9%). This was further aggravated with very low literacy rate of 1.1%, however with their intervention programs including trainings, the farmers possessed knowledge of pesticides as high as 88.7% [27]. In South India 42% of farmers responded to have good knowledge of pesticides and its use as majority (76%) of them was aware of training programs conducted by government [37]. Hence, farmer’s education through engagement with agricultural extension agents must be strengthened to increase our farmer’s knowledge and well understanding of the effects of pesticides in our country as well. More educations and trainings based on illustrations and videos must be presented to make both illiterates and literates understand the concept of pesticides and its risks. As even literate farmers have trouble interpreting texts and figures in pesticide labels.

Although this study is critical in investigating exposure of Bhutanese farmers to pesticides, there are limitations. Blood samples used in the test were only able to identify acute exposure to pesticides and do not have the capacity to assess the chronic burden of pesticides exposure the participants may have had. Therefore, boarder human health risk assessment through analysis of pesticides residues in different media such as food, drinking water, consumer products, soil and air should be conducted.

Since there are many pesticides used other than organophosphates and carbamates in the country this study was not be able to directly link the specific type of pesticides involved. Similarly, as pesticides affect different organs which are not specific to only the effects of pesticides and indirect exposure via food on target organ damage could not be directly linked.

## Conclusions

The findings from this study indicate that the sampled population of farmers had significant inhibition of AChE activity as compared to the unexposed group. This provides strong evidence of exposure to pesticides raising concerns on the health risk it poses. Farmers had a low level of knowledge and the practice was low. Poisoning risk factors identified; included random use and dosage of pesticides, poor management of pesticide waste, illicit import of pesticide across the border and inadequate knowledge on the purpose of pesticide application that may lead to high exposure and pesticide poisoning events in the future. Most of the farmers included in the study reported two or more clinical symptoms which were associated significantly with the depression of AChE enzyme activity. Pesticide usage in the country therefore needs to be controlled via regular monitoring of exposure, assessment of PPE usage and compliance, provide public awareness and advocacy on its health impacts.

## Supporting information

S1 Table Identification of poisoning risk factors. (DOCX)

S2 Table Knowledge assessment of farmers. (DOCX)

S3 Table Attitude assessment of farmers. (DOCX)

S4 Table Practice assessment of farmers. (DOCX)
