# Supplementary material for: Assessment of exposure to pesticides and the knowledge, attitude and practice among farmers of western Bhutan
Source: PLoS One. 2023 May 30;18(5):e0286348. doi: 10.1371/journal.pone.0286348 (PMC10228793; doi:10.1371/journal.pone.0286348)
Supplement: S1 Table — (DOCX) [file pone.0286348.s001.docx]

Supplement Table 1: Identification of poisoning risk factors

| **Risk factors** | **Variables** | **Frequency** | **%** | **Cum Percentage** | **Wilson 95% CI** | |
| --- | --- | --- | --- | --- | --- | --- |
|  |  |  |  |  | **Lower** | **Upper** |
| Spraying pesticides | Morning | 124 | 41.3 | 41.3 | 35.7 | 47.1 |
|  | Afternoon | 123 | 41.0 | 41.0 | 35.4 | 46.8 |
|  | Evening | 29 | 9.7 | 9.7 | 6.6 | 13.6 |
|  | Any time | 84 | 28.0 | 28.0 | 23.0 | 33.5 |
| Reasons for spraying pesticides | As routine regardless of whether I observe pests or not | 102 | 34.0 | 34.0 | 28.7 | 39.7 |
|  | When I see minor damage in my crop | 99 | 33.0 | 33.0 | 27.7 | 38.6 |
|  | When I see major damage in my crop | 7 | 2.3 | 2.3 | 0.9 | 4.8 |
|  | When my neighbors are spraying | 1 | 0.3 | 0.3 | 0.0 | 1.8 |
|  | Decide to spray after monitoring | 147 | 49.0 | 49.0 | 43.2 | 54.8 |
| Store of pesticides | Kitchen | 0 | 0.0 | 0.0 | 0.0 | 0.0 |
|  | Kitchen store | 3 | 1.0 | 1.0 | 0.2 | 2.9 |
|  | Farm Store | 180 | 60.0 | 60.0 | 54.2 | 65.6 |
|  | Toilet | 2 | 0.7 | 0.7 | 0.1 | 2.4 |
|  | Living room | 10 | 3.3 | 3.3 | 1.6 | 6.0 |
|  | Others* | 92 | 30.7 | 30.7 | 25.5 | 36.2 |
| Containers use while mixing pesticides | Kitchen utensils | 4 | 1.3 | 1.3 | 0.4 | 3.4 |
|  | Bathroom containers | 7 | 2.3 | 2.3 | 0.9 | 4.8 |
|  | Cattle feed containers | 10 | 3.3 | 3.3 | 1.6 | 6.0 |
|  | Designated containers for pesticides | 150 | 50.0 | 50.0 | 44.2 | 55.8 |
|  | Others | 129 | 43.0 | 43.0 | 37.3 | 48.8 |
| Management of empty pesticide containers | Discard in open | 13 | 4.3 | 4.3 | 2.3 | 7.3 |
|  | Reuse | 23 | 7.7 | 7.7 | 4.9 | 11.3 |
|  | Dispose with household garbage | 120 | 40.0 | 40.0 | 34.4 | 45.8 |
|  | Burn | 108 | 36.0 | 36.0 | 30.6 | 41.7 |
|  | Bury them in pit | 96 | 32.0 | 32.0 | 26.8 | 37.6 |
|  | Submit them to Agriculture officials | 3 | 1.0 | 1.0 | 0.2 | 2.9 |
| Pesticides source | Gewog/Dzongkhag office | 256 | 85.3 | 85.3 | 80.8 | 89.1 |
|  | Commission agent | 85 | 28.3 | 28.3 | 23.3 | 33.8 |
|  | Across the border | 13 | 4.3 | 4.3 | 2.3 | 7.3 |
|  | Neighbors | 3 | 1.0 | 1.0 | 0.2 | 2.9 |
|  | Individual vendors | 0 | 0.0 | 0.0 | 0.0 | 0.0 |
|  | Shops | 0 | 0.0 | 0.0 | 0.0 | 0.0 |
| Measurement of quantity for preparation of spray mixture | Do not use any measuring devices | 53 | 17.7 | 17.7 | 13.5 | 22.5 |
|  | Use the whole container | 50 | 16.7 | 16.7 | 12.6 | 21.4 |
|  | Use the container cap for liquid formulations | 193 | 64.3 | 64.3 | 58.6 | 69.8 |
|  | Tablespoons | 0 | 0.0 | 0.0 | 0.0 | 0.0 |
|  | Others | 52 | 17.3 | 17.3 | 13.2 | 22.1 |
| Discarding leftover pesticide mixture | Throw/Pour off in the field | 17 | 5.7 | 5.7 | 3.3 | 8.9 |
|  | Spray and finish whatever mixture have been made | 248 | 82.7 | 82.7 | 77.9 | 86.8 |
|  | Pour off in drains/irrigation canals | 1 | 0.3 | 0.3 | 0.0 | 1.8 |
|  | Store for reuse | 31 | 10.3 | 10.3 | 7.1 | 14.4 |
| Harvesting products after spraying pesticides | Same day | 0 | 0.0 | 0.0 | 0.0 | 0.0 |
|  | Next day | 29 | 9.7 | 9.7 | 6.6 | 13.6 |
|  | Within one week | 65 | 21.7 | 31.4 | 17.2 | 26.9 |
|  | Two weeks | 205 | 68.6 | 100.0 | 63.0 | 73.8 |
|  | Three weeks or more | 0 | 0.0 | 0.0 | 0.0 | 0.0 |
