# Supplementary material for: Assessment of exposure to pesticides and the knowledge, attitude and practice among farmers of western Bhutan
Source: PLoS One. 2023 May 30;18(5):e0286348. doi: 10.1371/journal.pone.0286348 (PMC10228793; doi:10.1371/journal.pone.0286348)
Supplement: S2 Table — (DOCX) [file pone.0286348.s002.docx]

Supplement Table 2: Knowledge on safe handling of Pesticides

| **Sl. No** | **Questions** | **Yes** | **No** | **Don’t Know** |
| --- | --- | --- | --- | --- |
|  |  | **n (%)** | **n (%)** | **n (%)** |
| 1 | Do you know how you get exposed to pesticides? | 177 (59.20) | 97 (32.44) | 25 (8.36) |
| 2 | Use of personal protective equipment (mask, gloves, apron, etc.) can prevent some of the health problems caused by the pesticides? | 271 (91.25) | 9 (3,03) | 17 (5.72) |
| 3^*^ | Do you receive any trainings on the pesticide storage, handling, transportation or formulations? | 59 (19.80) | 233 (78.19) | 6 (2.01) |
| 4 | Do you know about the use of different doses of pesticides for different crops? | 124 (41.75) | 140 (47.14) | 33 (11.11) |
| 5 | Is it good to spray pesticides when it is windy? | 43 (14.43) | 239 (80.20) | 16 (5.37) |
| 6 | I spray pesticides as a routine practice regardless of whether I see pests or not | 126 (42.28) | 167 (56.04) | 5 (1.68) |
| 7 | Do you think it is appropriate to mix different type of pesticides? | 67 (22.56) | 204 (68.69) | 28 (9.43) |

One point was deducted for each wrong answer, and one for right answer and selecting ‘don’t know’ answer did not affect the grade. *Question number 3 was excluded for overall knowledge assessment score.
