# Supplementary material for: Assessment of exposure to pesticides and the knowledge, attitude and practice among farmers of western Bhutan
Source: PLoS One. 2023 May 30;18(5):e0286348. doi: 10.1371/journal.pone.0286348 (PMC10228793; doi:10.1371/journal.pone.0286348)
Supplement: S3 Table — (DOCX) [file pone.0286348.s003.docx]

Supplement Table 3: Attitude on safe handling of Pesticides

| Sl. No | **Questions** | **Strongly disagree** | **Disagree** | **Neutral** | **Agree** | **Strongly Agree** |
| --- | --- | --- | --- | --- | --- | --- |
|  |  | **n (%)** | **n (%)** | **n (%)** | **n (%)** | **n (%)** |
| 1 | Using personal protective equipment is important while handling pesticides | 5 (1.67) | 0 (0.00) | 14 (4.67) | 56 (18.67) | 225 (75.00) |
| 2 | A cloth over your mouth and nose is enough for protection while handling pesticides | 98 (32.67) | 65 (21.67) | 35(11.67) | 58 (19.33) | 44 (14.67) |
| 3 | Clothes should be changed after spraying pesticides | 2 (0.67) | 7 (2.33) | 10 (3.33) | 65 (21.67) | 216 (72.00) |
| 4 | It is important to wash hands before eating/drinking while handling pesticides | 1 (0.34) | 0 (0.00) | 6 (2.02) | 39 (13.13) | 251 (84.51) |

One point was given for answering ‘agree/strongly agree’, and vice versa and being neutral did not affect the score.
