# Supplementary material for: Assessment of exposure to pesticides and the knowledge, attitude and practice among farmers of western Bhutan
Source: PLoS One. 2023 May 30;18(5):e0286348. doi: 10.1371/journal.pone.0286348 (PMC10228793; doi:10.1371/journal.pone.0286348)
Supplement: S4 Table — (DOCX) [file pone.0286348.s004.docx]

Supplement Table 4: Practice on safe handling of Pesticides

| Sl. No | **Questions** | **Never do this** | **Rarely do this** | **Occasionally do this** | **Often do this** | **Always do this** |
| --- | --- | --- | --- | --- | --- | --- |
|  |  | **n (%)** | **n (%)** | **n (%)** | **n (%)** | **n (%)** |
| 1 | I wear a mask when handling pesticides | 24 (8.05) | 11 (3.69) | 35 (11.74) | 51 (17.11) | 177 (59.40) |
| 2 | I wear gloves when handling pesticides | 40 (13.47) | 22 (7.41) | 37 (12.46) | 47 (15.82) | 151 (50.84) |
| 3 | I wash my hands after handling pesticides | 0 (0.00) | 2 (0.67) | 4 (1.35) | 31 (10.44) | 260 (87.54) |
| 4 | I change my clothes immediately after handling pesticides | 3 (1.01) | 6 (2.03) | 24 (8.11) | 46 (15.54) | 217 (73.31) |
| 5 | I take bath after handling pesticides | 13 (4.39) | 22 (7.43) | 52 (17.57) | 46 (15.54) | 163 (55.07) |
| 6 | I eat/chew doma/tobacco/smoke while handling pesticides | 200 (67.80) | 22 (7.46) | 33 (11.19) | 15 (5.08) | 25 (8.47) |
| 7 | I enter the field after spraying the pesticides | 201 (67.91) | 32 (10.31) | 30 (10.14) | 24 (8.11) | 9 (3.04) |
| 8 | I read pesticides labels before handling | 154 (52.38) | 23 (7.82) | 32 (10.88) | 37 (12.59) | 48 (16.33) |

One point was given for answering ‘agree’ and vice versa and occasionally do this did not affect the score.
